# A novel integrative and conjugative element in *Streptococcus pyogenes* carries a *cfr*-like gene associated with linezolid resistance in other species

**DOI:** 10.1128/msphere.00277-26

**Published:** 2026-08-21

**Authors:** Daniel B. Amusin, Lacy M. Simons, Dulce S. Garcia, Alan R. Hauser, Emma Office, Preeti Jaggi, David B. Williams, Larry K. Kociolek, Ami B. Patel, Stanford T. Shulman, Judith A. Guzman-Cottrill, Robert R. Tanz, Egon A. Ozer

**Affiliations:** 1 Department of Microbiology-Immunology, Northwestern University Feinberg School of Medicine 12244, Chicago, Illinois, USA; 2 Department of Medicine, Division of Infectious Diseases, Northwestern University Feinberg School of Medicine 12244, Chicago, Illinois, USA; 3 Department of Pediatrics, Division of Infectious Diseases, Emory University School of Medicine 12239, Atlanta, Georgia, USA; 4 Stanley Manne Children’s Research Institute, Ann & Robert H. Lurie Children’s Hospital of Chicago 2429, Chicago, Illinois, USA; 5 Department of Pediatrics, Northwestern University Feinberg School of Medicine 12244, Chicago, Illinois, USA; 6 Division of Pediatric Infectious Diseases, Ann & Robert H. Lurie Children’s Hospital of Chicago 2429, Chicago, Illinois, USA; 7 Department of Pediatrics, Division of Infectious Diseases, Oregon Health & Science University Doernbecher Children's Hospital 20240, Portland, Oregon, USA; 8 Division of Advanced General Pediatrics & Primary Care, Ann & Robert H. Lurie Children’s Hospital of Chicago 2429, Chicago, Illinois, USA; Medical College of Wisconsin, Milwaukee, Wisconsin, USA

**Keywords:** linezolid, oxazolidinones, *Streptococcus pyogenes*, cfr, Group A streptococcus

## Abstract

Treatment of severe invasive *Streptococcus pyogenes* infections has historically relied on β-lactam antibiotics with adjunctive clindamycin. Rising rates of lincosamide resistance have led to adoption of linezolid as an alternative adjunctive therapy. This report describes a clinical *S. pyogenes* isolate carrying a multi-drug resistance gene, *cfr*(C), known to confer the phenicol, lincosamide, oxazolidinone, pleuromutilin, and streptogramin A (PhLOPS_A_) resistance phenotype in other bacterial species. Strain GAS-390756 is a pharyngeal isolate collected from a child with pharyngitis in the United States. Whole-genome sequence analysis demonstrated that the isolate belonged to *emm* type 89 and carried an integrative and conjugative element with *erm*(B) and *cfr*(C) cargo genes (ICE*Spy*390756). Antimicrobial susceptibility testing demonstrated the isolate was resistant to erythromycin and clindamycin but susceptible to linezolid, consistent with the activity of *erm*(B) but not *cfr*(C). Comparative analysis of *cfr*(C)’s genomic context suggested that the promoter sequence may have been lost during ICE*Spy*390756’s acquisition of the gene. While GAS-390756 remains susceptible to linezolid, the expansion of *cfr*(C)’s host range into *S. pyogenes* and its presence on a novel mobile element are concerning. In light of known expression mechanisms for other silent antimicrobial resistance genes, this observation carries epidemiologic and public health importance.

**IMPORTANCE:**

Treatment of severe invasive *Streptococcus pyogenes* infection has traditionally relied on the adjunctive use of clindamycin with β-lactam antibiotic therapy. However, rising rates of clindamycin non-susceptibility have led clinicians to adopt linezolid as an alternative therapy. We identified *S. pyogenes* isolated from a child with pharyngitis that carried the *cfr*(C) gene. This gene causes resistance to multiple antibiotics, including clindamycin and linezolid, in other bacterial species. At the time of this study, we found no other instances of the *cfr*(C) gene carried by *S. pyogenes* in either published reports or the public genome sequence database. While this isolate remained sensitive to linezolid, the expanded host range of *cfr*(C) found in a novel potentially mobile genomic element may herald the eventual emergence of broader antibiotic resistance in *S. pyogenes*.

## OBSERVATION

*Streptococcus pyogenes* (group A streptococcus; GAS) is the most common cause of acute bacterial pharyngitis and an increasingly prevalent source of life-threatening invasive infections (iGAS), such as necrotizing fasciitis and streptococcal toxic shock syndrome (1). In addition to primary β-lactam therapy, the use of adjunctive clindamycin has been associated with favorable outcomes in the treatment of iGAS infection due to its toxin-suppressing and stationary phase activities (2). However, the United States Centers for Disease Control and Prevention have recently documented clindamycin resistance gene carriage in nearly 33% of iGAS isolates, and this represents a significant public health threat (1).

Linezolid is a last-resort antibiotic that targets the bacterial 23S ribosomal RNA (rRNA) and inhibits formation of a functional 70S ribosome. It has high potency against antibiotic-resistant, gram-positive pathogens, such as methicillin-resistant *Staphylococcus aureus*, vancomycin-resistant *Enterococcus,* and penicillin-resistant *Streptococcus pneumoniae* (3). Rising rates of clindamycin resistance coupled with linezolid’s favorable adverse-effect profile have led to the inclusion of linezolid as a non-inferior adjunct therapy for iGAS infection (4, 5). While GAS has remained universally susceptible to linezolid in global surveillance studies, multiple mechanisms of resistance have been identified in other species (6, 7). Among these, acquisition of *cfr* and *cfr*-like genes is of particular public health concern due to their association with mobile genomic elements (MGEs; plasmids, transposons, integrative and conjugative elements) and their ability to confer the phenicol, lincosamide, oxazolidinone, pleuromutilin, and streptogramin A (PhLOPS_A_ resistance) multi-drug resistance phenotype by methylation of 23s rRNA at position adenine A2503 (3, 8). This study describes an *S. pyogenes* isolate carrying a nonfunctional *cfr*-like gene.

Strain GAS-390756 is an *emm*89 oropharyngeal isolate collected by pharyngeal swab from a child with streptococcal pharyngitis in Atlanta, GA, USA, in 2022 (Supplemental Methods). Short-read whole-genome sequencing of the isolate was conducted as part of a multisite GAS genomic surveillance study, and the genome sequence was screened for carriage of antimicrobial resistance genes using AMRFinderPlus (Supplemental Methods) (9). *cfr*-like gene *cfr*(C) was identified within 500 bp downstream of the *erm*(B) macrolide, lincosamide, and streptogramin B (MLS_B_) resistance gene. To confirm the presence of *cfr*(C) and facilitate investigation of the genomic context of both resistance genes, genomic DNA was extracted from a new culture of isolate GAS-390756 and sequenced using Oxford Nanopore long-read sequencing. Hybrid short- and long-read assembly yielded a complete genome sequence consisting of a single circular chromosome (Supplemental Methods). The genome was annotated using the National Center for Biotechnology Information (NCBI) Prokaryotic Genome Annotation Pipeline, which identified a gene encoding a Cfr family 23S rRNA methyltransferase (10). Megablast alignment of the gene sequence against the core_nt database matched with >95% identity and >80% query coverage to isolates of *Clostridioides difficile*, *Clostridium perfringens*, *Faecalibacterium taiwanense*, *Nitratidesulfovibrio vulgaris*, *Bacteroides fragilis*, and *Campylobacter coli* (all other results demonstrated query coverage <13%). Alignment of the gene sequence of *cfr*(C) against the NCBI RefSeq, Whole Genome Shotgun (WGS), and nucleotide collection databases filtered for *S. pyogenes* yielded 0 matches in this species (11).

The *cfr*(C) gene has been characterized in several MGEs, including plasmids and integrative and conjugative elements (ICEs) (3, 12, 13). The genomic context of *cfr*(C) and *erm*(B) was investigated using a comparative genomics approach in which the complete genome of GAS-390756 was aligned to two complete *emm*89 genome sequences lacking *cfr*(C) in the RefSeq database (GCF_005164025.1, GCF_001039695.1). Both alignments demonstrated that *cfr*(C) and *erm*(B) of GAS-390756 were located within an identical 52.5 kb insertion (chromosome positions 568,681-621,161) flanked by a repeated 8 bp sequence (5′-TCCTAAGG-3′) within a tRNA gene. The guanine-cytosine (%GC) content across the inserted region was 46.0% compared to 38.8% across the GAS-390756 chromosome (Fig. S1), and annotation of the inserted region demonstrated a type IV secretion conjugation system, relaxase, and integrase, consistent with an ICE (ICE*Spy*390756; Table S1).

**Fig 1:**
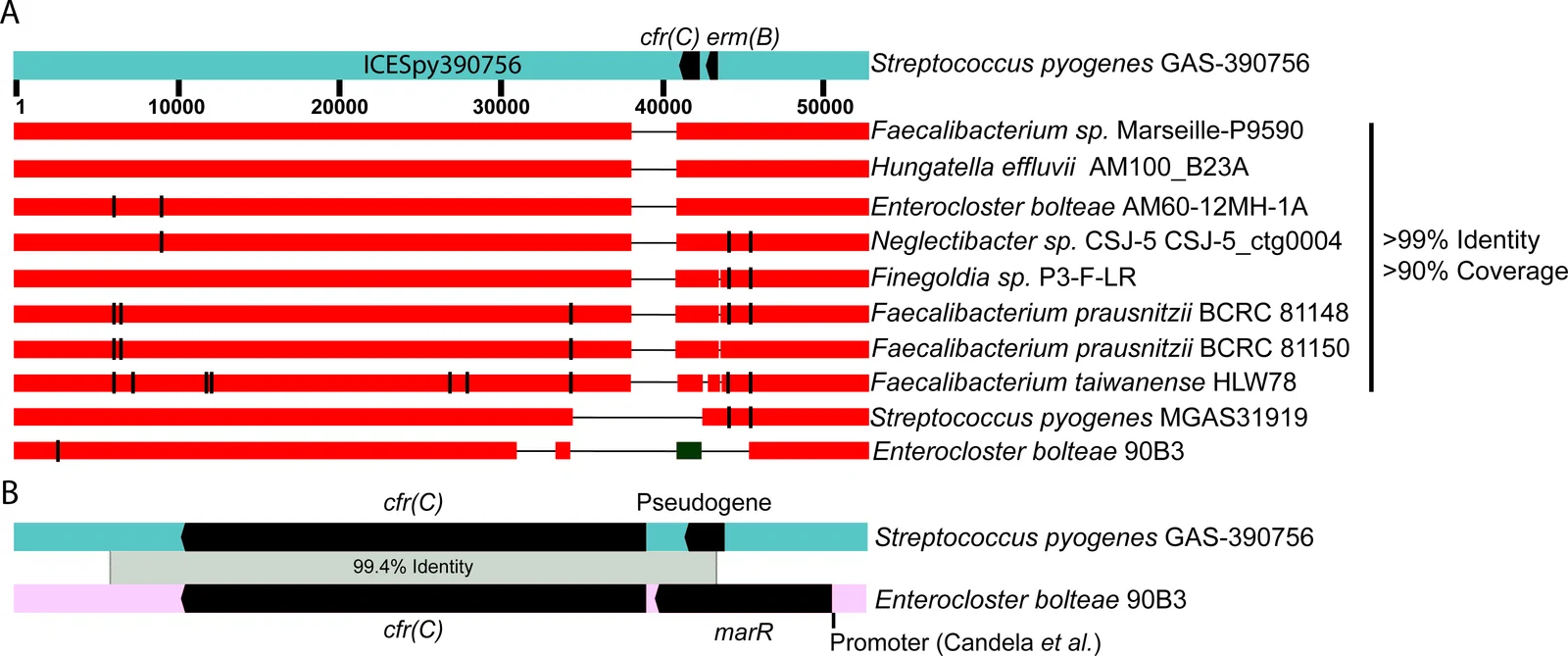
Alignment of ICE*Spy*390756 by BLAST to selected isolates from the NCBI Genome repository. (**A**) ICE*Spy*390756 is illustrated in blue. Eight alignments with >99% identity and >90% query coverage from the NCBI gram-positive RefSeq collection and nucleotide collection are shown in red. Alignments to selected GenBank isolates *S. pyogenes* MGAS31919 and *Enterocloster bolteae* 90B3 are also shown. Two contigs of *E. bolteae* 90B3 are represented in red and green. Vertical black lines indicate discontinuities in the alignments. (**B**) The *cfr*(C) containing region of ICE*Spy*390756 (blue) is aligned to the annotated genome of *E. bolteae* 90B3 (pink). The region of alignment with 99.4% identity is shown in gray. The promoter for *cfr*(C) and *marR* described by Candela et al. is marked by a line (12).

To investigate the possible origins of ICE*Spy*390756, the sequence was aligned to the NCBI nucleotide collection and RefSeq gram-positive collection using megablast (11). Eight sequences with >99% identity and 90% query coverage and carrying both *cfr*(C) and *erm*(B) were identified in the gram-positive *Faecalibacterium*, *Hungatella*, *Enterocloster*, *Neglectibacter*, and *Finegoldia* genera (Fig. 1A). Fifty-two other sequences had >99% identity and 80% query coverage; however, only three of these also carried *cfr*(C). Alignment of the ICE sequence against the NCBI WGS and RefSeq databases restricted to *S. pyogenes* sequences identified a single sequence (MGAS31919; GenBank DATVOD010000005.1) carrying *erm*(B) but not *cfr*(C) (Fig. 1A). In ICE*Spy*390756, *cfr*(C) was located directly upstream of transposition-associated genes *tnpA/tnpB/tnpC*. However, no nearby insertion sequences nor inverted repeats spanning *cfr*(C) were identified, and no sequences carrying the sequence spanning *tnpA* and *cfr*(C) were found in the NCBI nucleotide database. These results demonstrate that ICE*Spy*390756 is a novel ICE closely related to ICEs found in other gram-positive species. No evidence of ICE*Spy*390756 circular intermediates was detected in the whole-genome sequencing reads or by inverse PCR under routine growth conditions (Supplemental Methods).

Antimicrobial susceptibility to linezolid, erythromycin, and clindamycin was tested using the broth microdilution method. Comparison to Clinical and Laboratory Standards Institute (CLSI) reference standards demonstrated susceptibility to linezolid (MIC = 1 µg/mL) and resistance to erythromycin (MIC > 8 µg/mL) and clindamycin (MIC > 2 µg/mL). This result is consistent with the functional expression of *erm*(B) but not *cfr*(C) (14).

As the coding sequence of *cfr*(C) in GAS-390657 was identical to that of *cfr*(C) in other bacteria exhibiting linezolid resistance, we hypothesized that disruption of *cfr*(C)’s promoter is a possible mechanism by which *cfr*(C) is rendered nonfunctional. Candela et al. reported that *cfr*(C) was co-transcribed with a *marR*-like gene in *Enterocloster bolteae* (formerly *Clostridium bolteae*) strain 90B3 as part of ICE_90B3_ (12). BLAST alignment of ICE*Spy*390756 to the genome of *E. bolteae* 90B3 (RefSeq GCF_000371685.1) revealed alignment to two scaffolds. Accordingly, 74.4% of ICE*Spy*390756 aligned to scaffold 1.3 (98.8% identity; Fig. 1A in red). However, the region including *cfr*(C) aligned to a 1,551 bp sequence within scaffold 1.1 (99.4% identity; Fig. 1A in green), containing part of the *marR*-like gene in *E. bolteae* 90B3 but missing the upstream promoter sequence (Fig. 1B). These data suggest that while *E. bolteae* 90B3 contains sequences homologous to the ICE*Spy*390756 backbone, ICE*Spy*390756 is distinct from ICE_90B3_. The *cfr*(C) gene may be mobile and may have been separated from the promoter identified by Candela et al. However, further experimental evidence is needed.

GAS-390756 is the first reported *S. pyogenes* isolate to carry the multi-drug resistance genes *cfr*(C) and *erm*(B) on a novel ICE. Two variants of *cfr*(C) were first described by Tang et al. and Candela et al. in 2017 in *C. coli* (13), *C. difficile* (12), and *E. bolteae* (12). It has since been characterized in isolates from both clinical and zoonotic sources, including *C. perfringens* (15), *B. fragilis* (16), and now *S. pyogenes*. While this isolate of *S. pyogenes* remains susceptible to linezolid*,* the expansion of *cfr*(C)’s host range and evidence of possible gene mobility are concerning. Silent genes may form a reservoir for the development of antimicrobial resistance, and conversion to phenotypic resistance against other antibiotics has been described through mechanisms of insertion sequence acquisition or further genetic recombination (17). These findings support ongoing vigilance for the possible emergence of linezolid resistance in GAS.

## Data Availability

The complete genome assembly and sequencing reads of isolate GAS-390756 were deposited to NCBI under BioSample accession number SAMN54422403, GenBank accession number JBTKSW000000000, and Sequencing Read Archive (SRA) accession numbers SRR36663734 (Illumina reads) and SRR36663733 (Nanopore reads) under BioProject PRJNA817826.

## SUPPLEMENTAL MATERIAL

Supplemental material msphere.00277-26-s0001.docx Supplemental methods, table, and figure.

ASM does not own the copyrights to Supplemental Material that may be linked to, or accessed through, an article. The authors have granted ASM a non-exclusive, world-wide license to publish the Supplemental Material files. Please contact the corresponding author directly for reuse.
