# Supplementary material for: A novel integrative and conjugative element in Streptococcus pyogenes carries a cfr-like gene associated with linezolid resistance in other species
Source: mSphere. 2026 Aug 21;11(9):e00277-26. doi: 10.1128/msphere.00277-26 (PMC13621865; doi:10.1128/msphere.00277-26)
Supplement: Supplementary file 1 [file msphere.00277-26-s0001.docx]

**Supplemental Methods**

Isolate Collection

Isolate GAS-390756 was cultured from an oropharyngeal swab collected from a child with acute pharyngitis who tested positive for GAS using point-of-care antigen testing. The swab was struck onto 5% sheep blood agar and incubated at 35°C for 48 hours. A single beta-hemolytic colony was assessed for catalase activity, and the catalase negative sample was identified as GAS by the PathoDX latex agglutination test. The sample was sub-cultured onto tryptic soy agar/broth, and archived in 25% glycerol at -80°C.

Whole Genome Sequencing

To assemble the complete genome of GAS-390756, hybrid Illumina short-read and Oxford Nanopore long-read sequencing were performed. For short-read sequencing, GAS-390756 was sub-cultured and genomic DNA was extracted using the QIAamp DNA Mini Kit (QIAGEN) after incubation in proteinase K (New England Biolabs). The WGS library was constructed using the SeqWell ExpressPlex 2.0 kit and sequenced on an Illumina MiSeq instrument with the V3 sequencing kit yielding 300bp paired-reads. For long-read sequencing, DNA was extracted using the Qiagen EZ1&2 DNA Tissue Kit after incubation in lysozyme solution (50mg/mL). The Nanopore sequencing library was prepared using the Oxford Nanopore Native Barcoding Kit 24 with long-fragment buffer enrichment and sequenced on a GridION sequencer with an R10.4.1 flow cell for 72 hours, and basecalled with Dorado v7.11.2 using the super-accurate model (v5.2.0, 400bps) with minimum quality score of 10 (https://github.com/nanoporetech/dorado).

Bioinformatic Analysis

Illumina reads were trimmed using Fastp v0.24.0 with default parameters and assembled using SPAdes v4.0.0 with “automatic coverage cutoff” and “careful” parameters (1, 2). Contigs with average sequencing depth of less than 5 reads or shorter than 200bp were filtered from the assembly. Assembly quality was evaluated with CheckM v1.2.3 to have >99% completeness and <1% contamination (3).

Nanopore reads were randomly subsampled into 8 sets. Each set was assembled by Flye v2.9.6-b1802, Raven v1.8.3, and Miniasm v0.3-r179 polished by Minipolish v0.2.0 (4-7). All 24 assemblies were reconciled into a single consensus assembly using Autocycler v0.2.1 (8). The assembly was subsequently polished with nanopore reads using medaka v2.1.1 (https://github.com/nanoporetech/medaka) and Illumina reads using polypolish v0.6.1 and pypolca v0.4.0 (9, 10). Assembly quality was evaluated using CheckM v1.0.7 (3). Annotation of the assembly was performed using PGAP v6.9 (11).

The *emm* type of strain GAS-390756 was identified using the Centers for Disease Control and Prevention *Streptococcus* laboratory typing pipeline (<https://github.com/BenJamesMetcalf/GAS_Scripts_Reference>). Identification of antimicrobial resistance associated genes and mutations was performed using AMRFinderPlus 4.0.23 (12). *cfr*(C) and ICE*Spy*390756 presence in GAS and other species was performed by megablast search of respective sequences against the RefSeq, nucleotide, and WGS (filtered to *S. pyogenes*) databases of NCBI BLAST (13, 14).

The boundaries of the genetic element carrying the *cfr*(C) gene were identified by alignment of the complete genome of GAS-390756 to two *emm*89 complete genomes lacking *cfr*(C) obtained from NCBI RefSeq (GCF_001039695.1 and GCF_005164025.1) using the nucmer function from MUMmer v3.23 (15). The insertion site was identified by mapping the boundaries of this element to the annotations of both RefSeq assemblies. These annotations were downloaded directly from NCBI along with the RefSeq assemblies. To search for evidence of ICE*Spy*390756 circularization within WGS reads, both Illumina and Nanopore reads were aligned to the complete genome assembly using BWA MEM and minimap2 respectively (6, 16). The flanks of the ICE sequence were inspected for read soft clipping.

Search for insertion sequences and inverted repeats was performed using ISFinder BLAST (<https://www-is.biotoul.fr/blast.php>) and the einverted function from EMBOSS v6.6.0.0 using default parameters (17).

Inverse PCR

Inverse PCR was performed using MyTaq Red Mix (Meridian) and genomic DNA extracted from an overnight culture in tryptic soy broth to detect a circular extrachromosomal intermediate of ICE*Spy*390756. The predicted circular junction was targeted using two sets of outward-facing primers ~120 bp (F: 5’- AGGCGTGTGCTCTATCC-3’; R: 5’- CGGACGGTGTAAAATCCC-3’) and ~385 bp (F: 5’- GCTGATTAGCAGGAAGGG-3’; R: 5’- CTGCTTCGGCGGGTC-3’) from the predicted termini. Primers targeting *cfr*(C) were used as a positive control (F: 5’-AGCTCATAGCGGATATGCGC-3’; R: 5’-TTATTGGCATGAGCGTTTCCC-3’).

Antimicrobial Susceptibility Testing

Antimicrobial susceptibility testing for linezolid, erythromycin, and clindamycin was completed using the microbroth dilution method with tests run in duplicates. Quality control was ensured through parallel testing of the erythromycin, clindamycin, and linezolid sensitive strain *Streptococcus pneumoniae* ATCC 49619 and known erythromycin and clindamycin resistant strain *S. pyogenes* GAS-986663. Results were interpreted according to CLSI standard breakpoints (18).

**Supplemental Tables**

**Table S1. Annotated Sequence of ICESpy390756**

The sequence of ICE*Spy*390756 was annotated using the Prokaryotic Genome Annotation Pipeline (11). All positions are listed relative to the start of the ICE at position 568,681 on the chromosome of GAS-390756.

| Start | End | Strand | Product |
| --- | --- | --- | --- |
| 85 | 161 | + | tRNA-Arg |
| 731 | 934 | + | hypothetical protein |
| 931 | 1860 | + | DUF6017 domain-containing protein |
| 1867 | 2694 | + | ParA family protein |
| 2651 | 3577 | + | ParB/RepB/Spo0J family partition protein |
| 3586 | 4113 | + | hypothetical protein |
| 4168 | 4809 | + | DUF3846 domain-containing protein |
| 4820 | 5131 | + | DUF5720 family protein |
| 5168 | 5650 | + | PcfB family protein |
| 5650 | 7401 | + | VirD4-like conjugal transfer protein, CD1115 family |
| 7414 | 7734 | + | hypothetical protein |
| 7740 | 8132 | + | helix-turn-helix domain-containing protein |
| 8216 | 8431 | + | Maff2 family mobile element protein |
| 8457 | 9323 | + | VirB6/TrbL-like conjugal transfer protein, CD1112 family |
| 9341 | 9775 | + | PrgI family protein |
| 9735 | 12122 | + | VirB4-like conjugal transfer ATPase, CD1110 family |
| 12126 | 13862 | + | C40 family peptidase |
| 13877 | 14134 | + | DUF4315 family protein |
| 14124 | 14807 | + | DUF4366 domain-containing protein |
| 14959 | 16191 | + | ATP-binding protein |
| 16369 | 18444 | + | DNA topoisomerase 3 |
| 18441 | 19286 | + | LPD16 domain-containing protein |
| 19300 | 19896 | + | YodL domain-containing protein |
| 19893 | 20099 | + | transposon-transfer assisting family protein |
| 20118 | 28448 | + | LPD11 domain-containing protein |
| 28448 | 28780 | + | plasmid mobilization protein |
| 28897 | 29181 | + | hypothetical protein |
| 29244 | 30656 | + | relaxase/mobilization nuclease domain-containing protein |
| 30935 | 31804 | - | zinc metalloprotease HtpX |
| 31854 | 32117 | - | DUF1292 domain-containing protein |
| 32134 | 32388 | - | hypothetical protein |
| 32398 | 33438 | - | hypothetical protein |
| 33458 | 34276 | - | hypothetical protein |
| 34363 | 34707 | - | recombinase family protein |
| 34722 | 36422 | - | recombinase family protein |
| 36514 | 36660 | - | hypothetical protein |
| 36794 | 36994 | - | hypothetical protein |
| 37325 | 37747 | - | RNA polymerase sigma factor |
| 37989 | 39638 | - | IS66 family transposase TnpC |
| 39923 | 40282 | - | IS66 family insertion sequence element accessory protein TnpB |
| 40275 | 40655 | - | IS66 family insertion sequence element accessory protein TnpA |
| 40982 | 42232 | - | Cfr family 23S rRNA (adenine(2503)-C(8))-methyltransferase |
| 42334 | 42444 | - | topoisomerase DNA-binding C4 zinc finger domain-containing protein [pseudogene] |
| 42614 | 43351 | - | 23S rRNA (adenine(2058)-N(6))-methyltransferase Erm(B) |
| 43459 | 43842 | - | helix-turn-helix domain-containing protein |
| 43877 | 45013 | - | tyrosine-type recombinase/integrase |
| 45396 | 45773 | + | helix-turn-helix domain-containing protein |
| 45936 | 46490 | - | ImmA/IrrE family metallo-endopeptidase |
| 46542 | 46991 | - | helix-turn-helix domain-containing protein |
| 47278 | 47841 | + | hypothetical protein |
| 48104 | 48409 | + | helix-turn-helix transcriptional regulator |
| 48924 | 49331 | + | type II toxin-antitoxin system PemK/MazF family toxin |
| 49338 | 49574 | + | helix-turn-helix domain-containing protein |
| 49692 | 50315 | + | helix-turn-helix domain-containing protein |
| 50320 | 51693 | + | site-specific integrase |
| 51779 | 52084 | + | hypothetical protein |

**Supplemental Figures**

**Figure S1**. **G-C Content Along ICE*Spy*390756**

The percent G-C content along ICE*Spy*390756 is plotted using sliding windows of 500bp overlapping by 250bp. The average G-C content of ICE*Spy*390756 (46.0%) is represented by the blue horizontal line and the position of *cfr*(C) is highlighted in light blue.

**Appendix References**

1.	Chen S, Zhou Y, Chen Y, Gu J. 2018. fastp: an ultra-fast all-in-one FASTQ preprocessor. Bioinformatics 34:i884-i890.

2.	Prjibelski A, Antipov D, Meleshko D, Lapidus A, Korobeynikov A. 2020. Using SPAdes De Novo Assembler. Current Protocols in Bioinformatics 70:e102.

3.	Parks DH, Imelfort M, Skennerton CT, Hugenholtz P, Tyson GW. 2015. CheckM: assessing the quality of microbial genomes recovered from isolates, single cells, and metagenomes. Genome Res 25:1043-55.

4.	Kolmogorov M, Yuan J, Lin Y, Pevzner PA. 2019. Assembly of long, error-prone reads using repeat graphs. Nature Biotechnology 37:540-546.

5.	Vaser R, Šikić M. 2021. Time- and memory-efficient genome assembly with Raven. Nature Computational Science 1:332-336.

6.	Li H. 2016. Minimap and miniasm: fast mapping and de novo assembly for noisy long sequences. Bioinformatics 32:2103-2110.

7.	Wick RR, Holt KE. 2019. Benchmarking of long-read assemblers for prokaryote whole genome sequencing. F1000Res 8:2138.

8.	Wick RR, Howden BP, Stinear TP. 2025. Autocycler: long-read consensus assembly for bacterial genomes. Bioinformatics 41.

9.	Bouras G, Judd LM, Edwards RA, Vreugde S, Stinear TP, Wick RR. 2024. How low can you go? Short-read polishing of Oxford Nanopore bacterial genome assemblies. Microbial Genomics 10.

10.	Zimin AV, Salzberg SL. 2020. The genome polishing tool POLCA makes fast and accurate corrections in genome assemblies. PLOS Computational Biology 16:e1007981.

11.	Li W, O'Neill KR, Haft DH, DiCuccio M, Chetvernin V, Badretdin A, Coulouris G, Chitsaz F, Derbyshire MK, Durkin AS, Gonzales NR, Gwadz M, Lanczycki CJ, Song JS, Thanki N, Wang J, Yamashita RA, Yang M, Zheng C, Marchler-Bauer A, Thibaud-Nissen F. 2021. RefSeq: expanding the Prokaryotic Genome Annotation Pipeline reach with protein family model curation. Nucleic Acids Res 49:D1020-d1028.

12.	Feldgarden M, Brover V, Gonzalez-Escalona N, Frye JG, Haendiges J, Haft DH, Hoffmann M, Pettengill JB, Prasad AB, Tillman GE, Tyson GH, Klimke W. 2021. AMRFinderPlus and the Reference Gene Catalog facilitate examination of the genomic links among antimicrobial resistance, stress response, and virulence. Scientific Reports 11:12728.

13.	Zhang Z, Schwartz S, Wagner L, Miller W. 2000. A greedy algorithm for aligning DNA sequences. J Comput Biol 7:203-14.

14.	Morgulis A, Coulouris G, Raytselis Y, Madden TL, Agarwala R, Schäffer AA. 2008. Database indexing for production MegaBLAST searches. Bioinformatics 24:1757-64.

15.	Kurtz S, Phillippy A, Delcher AL, Smoot M, Shumway M, Antonescu C, Salzberg SL. 2004. Versatile and open software for comparing large genomes. Genome Biol 5:R12.

16.	Li H. 2013. Aligning sequence reads, clone sequences and assembly contigs with BWA-MEM. arXiv.

17.	Rice PM, Bleasby AJ, Ison JC. 2011. EMBOSS User's Guide: Practical Bioinformatics. Cambridge University Press.

18.	CLSI. 2025. Performance Standards for Antimicrobial Susceptibility Testing, 35th ed. CLSI supplement M100. Clinical and Laboratory Standards Institute.
